# Misplacement of left-sided double-lumen tubes into the right mainstem bronchus: incidence, risk factors and blind repositioning techniques

**DOI:** 10.1186/s12871-015-0138-1

**Published:** 2015-10-28

**Authors:** Jeong-Hwa Seo, Jun-Yeol Bae, Hyun Joo Kim, Deok Man Hong, Yunseok Jeon, Jae-Hyon Bahk

**Affiliations:** Department of Anesthesiology and Pain Medicine, Seoul National University Hospital, Seoul National University College of Medicine, 101 Daehak-ro, Jongno-gu, Seoul 03080 South Korea; Department of Anesthesiology and Pain Medicine, Severance Hospital, Yonsei University College of Medicine, 50-1 Yonsei-ro, Seodaemun-gu, Seoul 120-752 South Korea

**Keywords:** Airway management, Anesthesia, General, Bronchi, Intubation, Intratracheal, One-lung ventilation

## Abstract

**Background:**

Double-lumen endobronchial tubes (DLTs) are commonly advanced into the mainstem bronchus either blindly or by fiberoptic bronchoscopic guidance. However, blind advancement may result in misplacement of left-sided DLTs into the right bronchus. Therefore, incidence, risk factors, and blind repositioning techniques for right bronchial misplacement of left-sided DLTs were investigated.

**Methods:**

This was an observational cohort study performed on the data depository consecutively collected from patients who underwent intubation of left-sided DLTs for 2 years. Patients’ clinical and anatomical characteristics were analyzed to investigate risk factors for DLT misplacements with logistic regression analysis. Moreover, when DLTs were misplaced into the right bronchus, the bronchial tube was withdrawn into the trachea and blindly readvanced without rotation, or with 90° or 180° counterclockwise rotation while the patient’s head was turned right.

**Results:**

DLTs were inadvertently advanced into the right bronchus in 48 of 1135 (4.2 %) patients. DLT misplacements occurred more frequently in females, in patients of short stature or with narrow trachea and bronchi, and when small-sized DLTs were used. All of these factors were significantly inter-correlated each other (*P* < 0.001). In 40 of the 48 (83.3 %) patients, blind repositioning was successful.

**Conclusions:**

Smaller left-sided DLTs were more frequently misplaced into the right mainstem bronchus than larger DLTs. Moreover, we were usually able to reposition the misplaced DLTs into the left bronchus by using the blind techniques.

**Trial registration:**

ClinicalTrials.gov Identifier: NCT01371773.

## Background

For one-lung anesthesia during thoracic surgery, a left-sided double-lumen endobronchial tube (DLT) is preferred over a right-sided DLT because of its greater margin of safety for correct positioning in the left mainstem bronchus (LMB) [1]. After passing through the glottis and 90° counterclockwise rotation, the DLT is advanced into the LMB either blindly or by fiberoptic bronchoscopic (FOB) guidance. Although left-sided DLTs can be placed into the LMB more rapidly by a blind technique [2], it may be misdirected into the right mainstem bronchus (RMB) because the RMB has a larger internal diameter and diverges from the carina more vertically from the sagittal plane than the LMB [3, 4].

When left-sided DLTs are misdirected into the RMB, attempts to reposition the left-sided DLTs into the LMB usually fail without any manipulations [4], thus necessitating FOB guidance. However, FOBs may be inapplicable in emergency situations such as massive hemoptysis. Moreover, the smallest-caliber FOBs, which are necessary for small-sized DLTs, may not be always available or may be too fragile to guide DLTs [5]. Therefore, we devised a blind technique for redirecting into the LMB the left-sided DLTs that were misdirected into the RMB.

This observational cohort study assessed the incidence and risk factors of inadvertent RMB intubation of left-sided DLTs. Patients’ clinical and anatomical characteristics were analyzed to investigate the risk factors associated with the DLT misplacement. Additionally, the efficacy of the blind repositioning techniques was evaluated.

## Methods

### Study design and population

The study protocol was approved by Seoul National University Hospital Institutional Review Board (reference number H-1105-027-360) and was registered at ClinicalTrials.gov site (NCT01371773). This study was an observational cohort analysis performed on the data depository consecutively collected from patients who underwent one-lung anesthesia with left-sided DLTs for thoracic surgery from January 2009 to January 2011. Informed consents were waived owing to retrospective analysis of existing medical records and anonymous nature of the study. We excluded patients with intraluminal lesions in the mainstem bronchi and those who underwent tracheal intubation via methods other than direct laryngoscopy, such as using a FOB, video laryngoscopy or airway exchange catheter. Electronic medical records, chest radiography and computed tomography (CT) images of the enrolled patients were reviewed retrospectively.

### Left endobronchial intubation

The DLT size was selected according to patients’ gender and height [6]: a 39-Fr DLTs for males taller than 178 cm; a 37-Fr DLT for males taller than 160 cm and for females taller than 165 cm; a 35-Fr DLT for males shorter than 160 cm and for females 153–165 cm tall; and a 32-Fr DLT for females shorter than 153 cm. The 41-Fr DLTs are not used at our institution because we know from our clinical experience that they are not indicated for the Asians.

On arrival in the operating room, patients were monitored with electrocardiography, non-invasive blood pressure, and pulse oximetry. After placing a headrest under the patient’s head, general anesthesia was induced with fentanyl 1.0–1.5 mcg/kg, propofol 1.5–2.0 mg/kg and rocuronium 0.6–0.8 mg/kg; sevoflurane was used for anesthetic maintenance. Tracheal intubation was performed using a disposable polyvinyl chloride left-sided DLT (Mallinckrodt^TM^ endobronchial tube, Covidien, Minneapolis, MN, USA). Although we did not restrict intubation practitioners (anesthesia specialists or residents), all of them had learned the predetermined protocol of left-sided DLT placement and performed intubation under supervision of the experienced thoracic anesthesiologists. The DLT was initially inserted into the glottis with the bronchial tip oriented anteriorly under direct laryngoscopy. After the bronchial tip passed the vocal cords and the stylet was removed, the DLT was sufficiently rotated counterclockwise for directing the bronchial tip to the left. After verifying the left-sided direction of the bronchial tip, the DLT was advanced until slight resistance was encountered [7]. After the patient’s head was returned to the neutral position, LMB or RMB intubation was confirmed by direct vision of a FOB (LF-DP or LF-GP, Olympus Optical Co., Tokyo, Japan), and depth of the DLT was correctly adjusted. If the DLT was correctly placed in the LMB, the subsequent anesthetic management was performed at the attending anesthesiologists’ discretion.

### Blind repositioning method

If DLTs had been misplaced into the RMB, both the tracheal and bronchial cuffs were deflated and the DLT was withdrawn into the mid-trachea. Then, the protocol for blindly redirecting DLTs into the LMB was applied step-by-step as follows by the two thoracic anesthesiologists (JHS or JHB): (1) The DLT was advanced to the pre-determined depth while turning the patient’s head to the right without rotating the DLT; (2) If the DLT was re-entered into the RMB, it was advanced while turning the patient’s head to the right with the DLT 90° counterclockwise rotated. (3) If the DLT was misplaced again into the RMB, it was readvanced while turning the patient’s head to the right with the DLT 180° counterclockwise rotated. If all the blind methods failed, the DLT was guided into the LMB using a FOB. All the procedure was performed while supplying 100 % oxygen and each step lasted no more than 10 s.

### Risk factors for right endobronchial misplacement

**Fig. 1:**
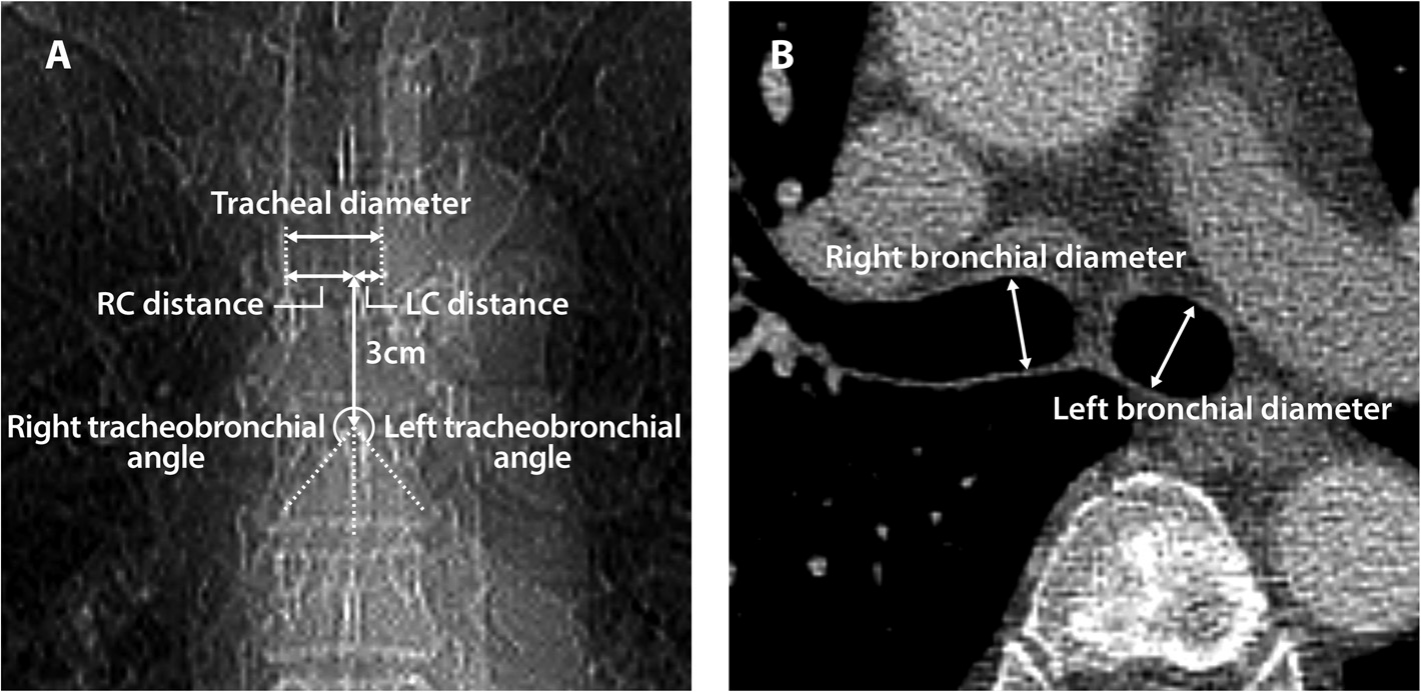
Anatomical variables that were measured on the plain chest radiography (**a**) and computed tomography (**b**)

To identify anatomical risk factors for inadvertent RMB intubation of the left-sided DLTs, several parameters were measured on plain chest radiography and CT images. An investigator (HJK), who was unaware of the study protocol and had undergone specific bedside training by a thoracic radiologist, conducted all the measurements on a 21-inch monitor of Picture Archiving and Communication System (M-view^TM^, Marotech Inc., Seoul, Korea) using electronic calipers and protractors that were incorporated into the system. On the chest radiography images, after drawing a vertical line from the carina, the horizontal distances between the line and the right and left internal borders of the trachea (RC and LC distance, respectively) were measured respectively at 3 cm above the carina (Fig. 1a). Because the vertical length of the angulated bronchial tip of DLTs is about 3 cm, the RC and LC distances were measured at 3 cm above the carina. The tracheal diameter was calculated as the sum of the RC and LC distances. The angles between the long axis of the trachea and one of either the RMB or LMB were also measured, respectively (Fig. 1a).

In addition, chest CT images with section thicknesses 1–3 mm were used to measure the internal diameters of the RMB and LMB. The images were displayed at the mediastinal window (400 Hounsfield units of width and 25 Hounsfield units of level) with an one-on-one format. The bronchial diameters were measured at a plane about 1.5 cm below the carina and where each mainstem bronchi was respectively seen as a singular structure. Each mainstem bronchial diameter was measured perpendicular to the portion of the bronchus that was parallel between the anterior and posterior walls of the bronchus (Fig. 1b) [8].

The primary outcomes of this study were the incidence and the clinical or anatomical risk factors of inadvertent RMB intubation of the left-sided DLTs. The secondary outcome is the success rate of the aforementioned blind repositioning technique.

### Statistical analysis

Continuous variables were tested for a normal distribution using the Kolmogorov-Smirnov test, and parametric and non-parametric data were expressed as mean ± SD and median (IQR), respectively. Categorical variables were presented as the number of patients (%). Various parameters were compared using paired or unpaired t-tests and Fisher’s exact test as appropriate. To identify risk factors for RMB misplacement of left-sided DLTs, logistic regression analysis was performed and their odds ratios with a 95 % confidence interval were calculated. The interrelationships among the potential risk factors were identified using correlation analysis with Pearson’s or Spearman’s correlation coefficient. Statistical Package for Social Sciences software (version 18.0, SPSS Inc., Chicago, IL, USA) was used for statistical analysis. All reported *P*-values were two-sided and *P* < 0.05 was considered to indicate statistical significance.

## Results

**Fig. 2:**
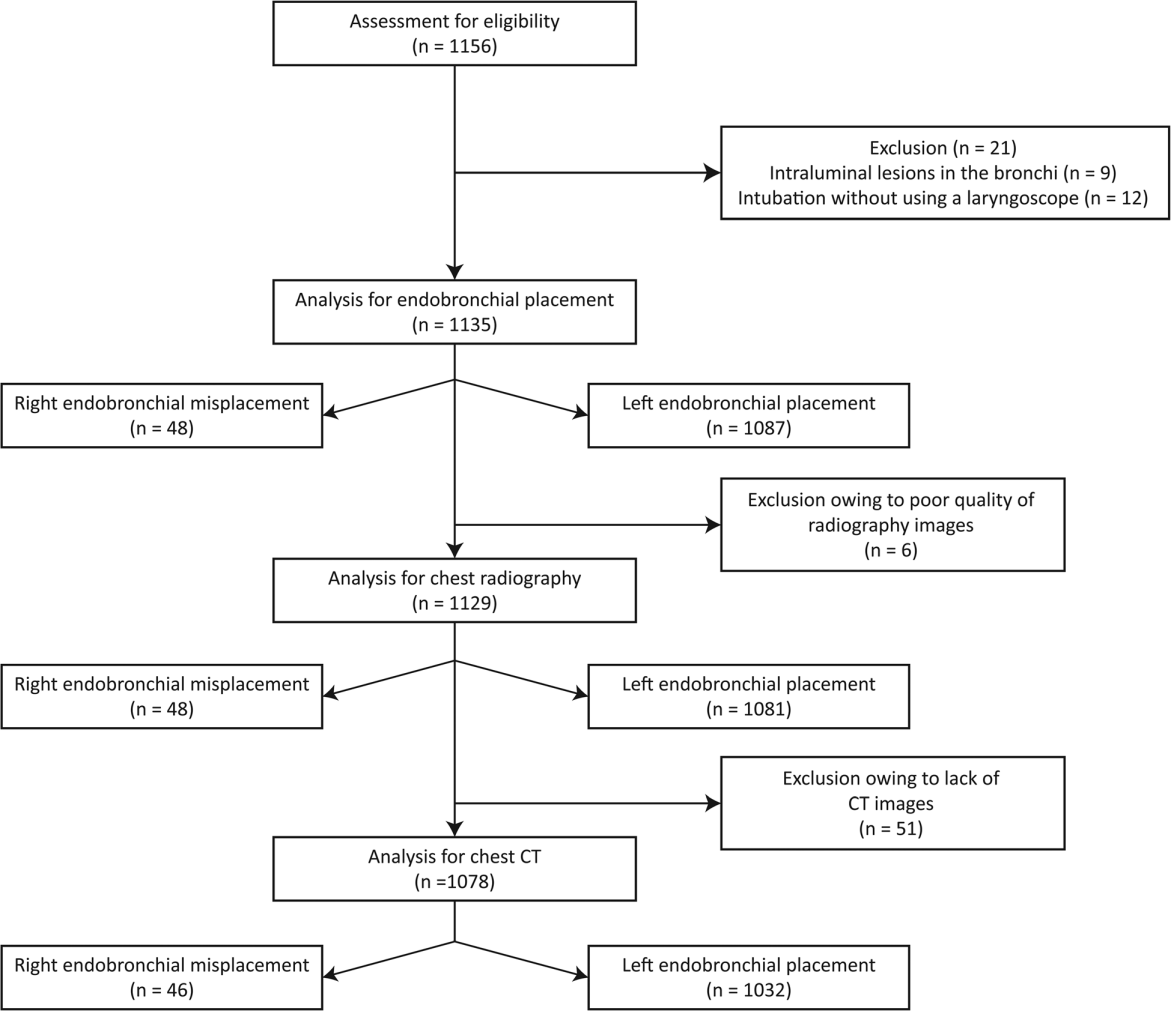
Flow diagram of the study. CT: computed tomography

**Fig. 3:**
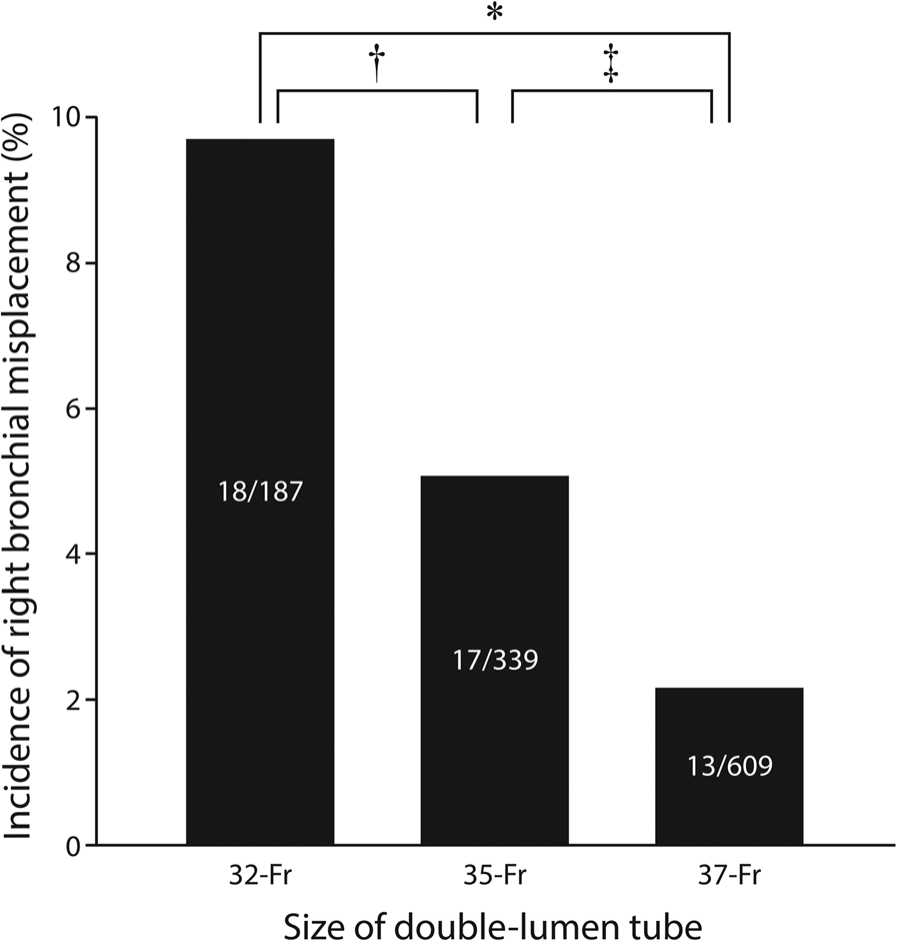
Incidence of right bronchial misplacement depending on size of double-lumen tubes. *P < 0.001 for 32-Fr *vs.* 37-Fr. ^†^ *P* = 0.046 for 32-Fr *vs*. 35-Fr. ^‡^ *P* = 0.02 for 35-Fr *vs.* 37-Fr

A total of 1156 patients underwent one-lung anesthesia with left-sided DLTs for the study period of 2 years. After exclusion of 21 patients (Fig. 2), the remaining 1135 patients constituted the study cohort (Table 1). Left-sided DLTs were inadvertently advanced into the RMB in 48 patients (4.2 %). Right bronchial misplacement occurred more frequently in females, in patients with short stature or narrower tracheal and bronchial diameters, and in those who received smaller DLTs (Table 1 and Fig. 3).

**Table 1:** Clinical and anatomical characteristics of the patients

	Right bronchial misplacement (*n* = 48)	Left bronchial placement (*n* = 1087)	*P*-value
Age (yr)	60 ± 12	57 ± 15	0.181
Gender (male/female)	15/33	687/400	<0.001
Height (cm)	159 ± 8	164 ± 8	<0.001
Weight (kg)	60 ± 11	61 ± 10	0.884
Laryngoscopic grade (1/2/3)	41/5/2	920/130/37	0.887
Double-lumen tube size (32/35/37 Fr)	18/17/13	169/322/596	<0.001
Level of intubation practitioner^a^ (2nd/3rd/4th/specialist)	22/11/9/6	525/271/189/102	0.833
RC distance (mm)	7.3 ± 3.7	8.1 ± 3.0	0.136
LC distnace (mm)	6.7 ± 3.4	7.4 ± 2.8	0.122
Tracheal diameter (mm)	14.0 ± 2.2	15.4 ± 2.6	0.001
Right tracheobronchial angle (°)	147 ± 11	146 ± 11	0.456
Left tracheobronchial angle (°)	138 ± 13	137 ± 11	0.496
Right bronchial diameter (mm)	12.0 ± 2.0	13.0 ± 1.9	0.003
Left bronchial diameter (mm)	10.7 ± 1.7	11.4 ± 1.8	0.016

Values are given as mean ± SD or number of patients

RC and LC distances: horizontal distances between the carina and the right or left internal borders of the trachea at 3 cm above the carina; Tracheal diameter: sum of the RC and LC distances

^a^The level of experience of the anesthesiologists performing the first intubation attempt. The ordinal numbers mean the grades of anesthesia residents

The univariate logistic regression analysis showed that six parameters--gender, height, DLT size, tracheal diameter, right and left bronchial diameters--were significantly associated with DLT misplacements into the RMB (Table 2). However, because all of the six parameters were significantly inter-correlated in the correlation analysis (*P* < 0.001), the multivariate logistic regression analysis was not performed.

**Table 2:** Logistic regression analysis for right bronchial misplacement of left-sided double-lumen tubes

	Regression coefficient (SE)	Odds ratio (95 % CI)	*P*-value
Age	0.01 (0.01)	1.01 (0.99 to 1.04)	0.182
Female	1.33 (0.32)	3.78 (2.03 to 7.04)	<0.001
Height	−0.07 (0.02)	0.93 (0.89 to 0.97)	<0.001
Weight	−0.01 (0.01)	1.00 (0.97 to 1.03)	0.884
Laryngoscopic grade^a^			
2	−0.15 (0.48)	0.86 (0.34 to 2.22)	0.760
3	0.19 (0.74)	1.21 (0.28 to 5.21)	0.795
Double-lumen tube size^b^			
32-Fr	1.59 (0.37)	4.88 (2.35 to 10.17)	<0.001
35-Fr	0.88 (0.38)	2.42 (1.16 to 5.05)	0.018
RC distance	−0.08 (0.05)	0.93 (0.84 to 1.02)	0.136
LC distnace	−0.08 (0.05)	0.92 (0.83 to 1.02)	0.122
Tracheal diameter	−0.22 (0.06)	0.81 (0.71 to 0.91)	0.001
Right tracheobronchial angle (°)	0.10 (0.07)	1.01 (0.98 to 1.04)	0.456
Left tracheobronchial angle (°)	0.01 (0.08)	1.01 (0.98 to 1.04)	0.495
Right bronchial diameter (mm)	−0.21 (0.07)	0.81 (0.70 to 0.93)	0.003
Left bronchial diameter (mm)	−0.20 (0.08)	0.82 (0.70 to 0.97)	0.017

RC and LC distances: horizontal distances between the carina and the right or left internal borders of the trachea at 3 cm above the carina; Tracheal diameter: sum of the RC and LC distances

^a^Laryngoscopic grade 1 and ^b^Size of 37-Fr were considered as the reference value for the analysis

Moreover, among the 48 patients, who had their DLTs misdirected into the RMB, the DLTs were successfully repositioned into the LMB by the blind techniques in 40 patients (83.3 %) (Table 3). The success rate of repositioning was higher in the second step (68 %) than in the first (23 %) or third steps (33 %). There were no adverse events such as hypoxemia or accidental extubation during repositioning of DLTs. Furthermore, postoperative severe airway complications were not observed in the patients who had underwent the blind repositioning of DLTs.

**Table 3:** Blind repositioning of the misplaced left-sided double-lumen tubes into the left mainstem bronchus

Protocol	Incidence of successful repositioning	Cumulative incidence of successful repositioning	DLT size (32/35/37 Fr)
Turning head to the right without rotating DLT	11/48 (23 %)	11 (23 %)	2/5/4
Turning head to the right with rotating DLT 90° counterclockwise	25/37 (68 %)	36 (75 %)	10/9/6
Turning head to the right with rotating DLT 180° counterclockwise	4/12 (33 %)	40 (83 %)	2/1/1
Using fiberoptic bronchoscopy	7/8 (88 %)	47 (98 %)	3/2/2
Failed to reposition DLT	1		1/0/0

Values are expressed as number of patients (%). Right bronchial misplacement occurred in 48 of 1135 patients

*DLT* left-sided double-lumen endobronchial tube

## Discussion

If left-sided DLTs are misplaced into the RMB, orifice of the right upper lobar bronchus shall be blocked inhibiting ventilation or collapse of the right upper lobe during one-lung anesthesia [9]. Therefore, since right bronchial misplacement of left-sided DLTs is a critical adverse event that disturbs successful OLV, identifying patients with higher risks of the DLT misplacement is important. In our study, female, short stature, smaller DLTs, narrower tracheal and bronchial diameters were significantly associated with RMB misplacement of left-sided DLTs.

Several anatomical characteristics of the trachea and bronchi, measured on the routine preoperative imaging tests, were hypothesized to be potential risk factors of the DLT misplacement. The location of the carina, namely how the trachea diverges into the mainstem bronchi, was thought to affect the direction of DLT advance into the mainstem bronchi. Moreover, the tracheobronchial angles were also thought to affect the direction of the intubated bronchus. However, RMB misplacement of the left-sided DLTs appeared to be associated with narrower tracheal and bronchial diameters rather than relative position of the carina or tracheobronchial angles.

Generally, female patients of short stature have narrower tracheal and bronchial diameters, and undergo tracheal intubation with smaller DLTs [10]. Moreover, gender, height, tracheal and bronchial diameters, and DLT size were significantly inter-correlated. The left-sided DLT could be successfully advanced into the LMB because its curved bronchial tip faces to the left following counterclockwise rotation. However, even after turning the DLTs left, smaller DLTs may continue to slide into the RMB because the RMB has a larger internal diameter and diverges more vertically from the trachea than the LMB [3, 4]. Besides, the bronchial tip angle of 32-Fr left-sided Mallinckrodt™ DLTs appears to be more obtuse than those of larger-sized DLTs (153–155° for 32-Fr DLTs *vs*. 147–150° for 35-, 37-, and 39-Fr DLTs: authors’ measurements). Therefore, the use of small-sized DLTs seems to be more influential for RMB misplacement rather than any other demographic or anatomical factors, as described in the previous studies [11, 12].

FOBs are indispensable for repositioning the misplaced DLTs, but it may not be always available. For 32- or 35-Fr DLTs, which are commonly selected for the Asian females of short stature [10], only smallest-caliber FOBs can be accommodated. Because the FOB may be too fragile to guide the relatively stiff DLT towards the intended direction in an our experience [5], the bronchial tip of the DLT could not be guided into the LMB after several attempts, resulting in completely breaking the FOB.

Once a left-sided DLT is misplaced into the RMB, redirecting the DLT into the LMB may continue to fail without any manipulations [4]. Therefore, in order to increase the chance of correct positioning, two techniques were adopted. First, turning the patient’s head to the right is known to facilitate the left bronchial insertion of rigid bronchoscopes or single-lumen endotracheal tubes [4, 13]. The most likely reason is that turning the head to the right shifts the larynx to the same direction in relation to the carina, thereby aligning the axis of LMB with that of trachea and providing a straighter pathway into LMB [4]. Second, counterclockwise rotation of single-lumen endotracheal tubes is also known to facilitate left bronchial intubation [13]. During blind advancement of left-sided DLTs through the trachea, the proximal part of DLTs is rotated 90° counterclockwise in order to turn left the curved bronchial tip [14]. However, if the distal part of DLTs is engaged somewhere below the glottis, its rotation may be still insufficient. Because we speculated that insufficient rotation of DLTs could be one of the factors for RMB misplacement, a 180° rotation was suggested as the third step. Moreover, because the first step seemed less effective than the second and third steps, we may recommend right-side turning of the head in conjunction with simultaneous counterclockwise rotation of the DLT as a first attempt to minimize the numbers of repositioning attempts.

This study had inherent limitations owing to its observational cohort analysis. However, the data was consecutively collected according to the predetermined protocol, thus there was no missing data of the right bronchial misplacement during the study period. Moreover, the blind repositioning techniques may lead to airway injuries, but we did not directly evaluate any airway lesions and clinical complaints such as sore throat and hoarseness. Although any airway complications were not observed except for minor subjective complaints, its usability may not be transferable to less experienced anesthesiologists. Furthermore, designing a prospective randomized trial regarding the step-by-step efficiency of the blind repositioning techniques would be almost unlikely because RMB misplacement of the left-sided DLTs is relatively uncommon. Lastly, the overall incidence of the RMB misplacement seemed relatively low in our study as compared with the previous studies [12, 15], which might be due to ethnic difference, retrospective design or the fact that this study was performed in a high-volume facility of thoracic cases. Further studies may be required to investigate this issue.

## Conclusions

In conclusion, RMB misplacement of the left-sided DLTs occurred more frequently in females, in patients with short stature or narrow tracheal and bronchial diameters, and in those who received smaller DLTs. Therefore, anesthesiologists should take into account the higher possibility of RMB misplacement when using small left-sided DLTs. Besides, when FOBs are unavailable, this blind techniques, which consist of right-side turning of the head and counterclockwise rotation of the DLTs, can be useful for repositioning the misplaced DLTs.

## Abbreviations

DLT: Double-lumen endobronchial tube
LMB: Left mainstem bronchus
FOB: Fiberoptic bronchoscopy
RMB: Right mainstem bronchus
CT: Computed tomography
